# Robot-assisted retroperitoneal lymphadenectomy (RPLND): video case report

**DOI:** 10.1590/S1677-5538.IBJU.2020.0828

**Published:** 2021-01-10

**Authors:** Daniel C. Gomes, Walter H. Da Costa, Éder S. Brazão, Eugênio R. C. Borges, Lucas B. Vergamini, Bruno V. Ricci, Stênio C. Zequi

**Affiliations:** 1 AC Camargo Cancer Center Núcleo de Urologia São PauloSP Brasil Núcleo de Urologia, AC Camargo Cancer Center, São Paulo, SP, Brasil.

## Abstract

**Introduction and Objective:**

Germ cell tumors account for 90 to 95% of all testicular tumors. Approximately one-half of these cases are seminomas, and the other half constitutes non-seminomatous germ cell tumors (NSGCTs). The standard of care for men with more advanced or disseminated NSGCTs (stage IIB or higher) is to administer chemotherapy. In general, any patient with one or more residual retroperitoneal lymph nodes larger than 1cm following chemotherapy should undergo RPLND (1, 2). Laparoscopic/robotic RPLND is a technique that may reduce morbidity compared to the classic procedure (3). AFP, HCG and LDH are important tumor markers that are helpful in diagnosis, staging and evaluation of response to the therapy, although recently publications suggest that neutrophil to lymphocyte ratio (NLR) could be used as an alternative marker (4, 5).

**Materials and Methods:**

We present a case of a 27-year-old male with prior right orchiectomy for NSGCT and residual retroperitoneal mass of 3.9 x 2.3cm (pT1pN2M0S1 EC IIB-low risk IGCCCG). He underwent platin-based chemotherapy and was eligible for surgery. Robot-assisted RPLND was then proceeded.

**Results:**

Console operative time of 90min, total blood loss of 60cc and hospital discharge within 24h. Pathology report showed a post-pubertal teratoma metastasis and absence of capsular extravasation in 3 lymph nodes dissected. The control CT-Scan showed a significant improvement in radiological pattern and the patient is still on follow-up until the date of this video publication.

**Conclusions:**

The capability for 3-D visualization and complex dissection of vascular structures makes the robotic platform a powerful ally. Although further studies are required, our initial experience suggests that the robotic RPLND is feasible and reproducible, and should be encouraged in centers with high cancer volume where the robotic platform is available.

